# Genome description of a potentially novel species of Billgrantia sp. strain SL18_1 isolated from Sambhar Lake, India

**DOI:** 10.1099/acmi.0.001093.v3

**Published:** 2026-06-25

**Authors:** Chakresh Kumar, Anwesha Ghosh, Prasanta Sanyal, Punyasloke Bhadury

**Affiliations:** 1 Integrative Taxonomy and Microbial Ecology Research Group, Department of Biological Sciences, Indian Institute of Science Education and Research Kolkata, Mohanpur-741246, Nadia, India; 2 Centre for Climate and Environmental Studies, Indian Institute of Science Education and Research Kolkata, Mohanpur-741246, Nadia, India; 3 Stable Isotope Laboratory of IISER Kolkata (SILIKA), Department of Earth Sciences, Indian Institute of Science Education and Research Kolkata, Mohanpur-741246, Nadia, India

**Keywords:** *Billgrantia*, denitrification, nitrogen cycling, osmoregulation, Sambhar Lake

## Abstract

A potentially novel species belonging to the genus *Billgrantia* bearing strain number SL18_1 has been isolated from Sambhar Lake, the largest inland salt water wetland located in the north of India. Based on 16S rRNA sequence search in International Nucleotide Sequence Database Collaboration, the isolate showed 99.3% identity with *Billgrantia chromatireducens*. Based on long-read sequencing using Oxford Nanopore Technologies chemistry, followed by annotation, the genome size of this organism has been found to be ~4.14 Mb. The genome has a G+C content of 66.27 mol% and is closest to *Billgrantia campisalis* based on genome relatedness. The functional annotation of draft genome has revealed the presence of key genes involved in nitrate reduction and denitrification pathways, including *nirB*, *nirD* and *nasD* (nitrate reductase), *nirS* (nitrite reductase) and *norB* and *norC* (nitric-oxide reductase). The *nosZ* coding for nitrous-oxide reductase has been also identified from the draft genome. This strain also possesses genes linked to the breakdown of benzoate compounds as well as osmoregulatory genes, which can possibly allow the bacterium to adapt to high osmotic stress. Besides, the presence of *ibaG* gene within the genome may allow this organism to adapt to acidic stress. Genes linked to multidrug resistance, including *mdtA*, *mdtB* and *mdtK*, have been identified within the genome of *Billgrantia* sp. strain SL18_1.

## Data Summary

The whole-genome sequence data are available in NCBI with BioProject Accession Number PRJNA1287240 and BioSample Accession Number SRR34440939.

## Introduction

The genus *Billgrantia*, first described in 2023, is a member of the class *Gammaproteobacteria*, order *Oceanospirillales* and the family *Halomonadaceae* [1,3]. The *Halomonadaceae* is the largest family harbouring halophilic bacteria and is represented by more than 160 valid species [1,4]. Many strains of this family have been isolated from salt lakes, soda lakes, salty soil, solar salterns, deep-sea hydrothermal vents, sea ice, lake silt, seafood, marine invertebrates, effluents and other saline habitats [5]. The family has undergone several reclassifications in the last few years. Recently, de la Haba *et al.* [1] suggested a revision in the family suggesting the splitting of previously described genus *Halomonas* into seven new genera, *Halomonas sensu stricto*, *Vreelandella*, *Billgrantia*, *Franzmannia*, *Litchfieldella*, *Onishia *and *Modicisalibacter*. Because of wide metabolic and physiological adaptations, the halophilic bacteria can have promising applications in industrial biotechnology because of their ability to produce exopolysaccharides, polyhydroxyalkanoates and compatible solutes; can degrade aromatic compounds as well as oxidize Fe(II) and Mn(II); function as denitrifiers and more [3,6].

To thrive in saltwater environments, halophilic microbes need to counteract an osmotic gradient across their membrane. Complementary solutes are organic substances that accumulate intracellularly to achieve this adaptation [7,8]. Complementary solutes are accumulated by cells either through environmental absorption or production. Although uptake is a more energetically advantageous choice [9,10], the environment does not always provide right solutes or precursor chemicals. Changing environmental circumstances [9] influence transport systems, proteins and enzymes that catalyse the uptake and *de novo* synthesis of suitable solutes, determining capacity of an organism to endure salinity extremes.

## Methods

The surface water sample was collected from Sambhar Lake, a Ramsar wetland of International Importance, located in the Jaipur district of Rajasthan, India (26° 54′ 40″ N 75° 10′ 36″ E). The sample was collected in a 50 ml sterile centrifuge tube (Tarsons, India) using aseptic method and immediately transported to the laboratory under controlled temperature conditions. Briefly, ~20 µl of the collected surface water sample was inoculated to Luria-Bertani agar plates with standard composition of 1% tryptone, 1% NaCl and 0.5% yeast extract. These plates were incubated at 37 °C overnight in order to mimic the environmental conditions that existed at the time of sampling. A pure bacterial isolate, designated as SL18_1, was subsequently obtained through successive steps of re-streaking. A bright field microscope (Bx53, Olympus, Japan; 1,000× magnification) was used to analyse the isolated strain SL18_1 in order to ascertain its cell shape and Gram stain characteristics. Genomic DNA (gDNA) was extracted from the culture that had grown overnight using a modified version of the phenol-chloroform method [11,12]. The quality and quantity of the gDNA were ascertained following published protocol. For undertaking phylogeny based on 16S rRNA, FC27 and RC1492 primers [13] were used for PCR, followed by Sanger di-deoxy sequencing. The nucleotide sequences of the primers are provided in Table S1 (available in the online Supplementary Material). Whole-genome sequencing was undertaken in a MinION platform using Oxford Nanopore Technologies sequencing chemistry following published protocols (e.g. detailed in [14]). Porechop (v0.2.0) was used to remove adapters from long reads, and NanoPlot was used to assess quality of the data [15]. To construct the *de novo* genome, Flye (v2.9.3 [16]) was applied. CheckM [17] was used to quantify contamination and genomic completeness. The draft genome was annotated using Prokka (v1.14.6 [18]). The quality of assembled genome was verified using QUAST (v5.2.0 [19]) and CheckM [17]. Genome map was visualized using Proksee [20]. For *in silico* phenotyping, Traitar was used (https://github.com/hzi-bifo/traitar[21]). Whole-genome sequence-based phylogeny was undertaken using the type (strain) genome server (TYGS) (https://tygs.dsmz.de) [22 23]. Genome distance was measured by TYGS using the genome blast distance phylogeny. FASTME 2.1.4 was used to infer the evolutionary tree with branch support based on intergenomic distances [24]. MiGA was implemented to perform average amino acid identity (AAI), taxonomic classification and presence of essential genes (https://aau.microbial-genomes.org/ [25]). Biosynthetic gene clusters (BGCs) encoding natural products or secondary metabolites were identified using AntiSMASH [26]. The EZBioCloud server was used to compute the average nucleotide identity (ANI) (https://www.ezbiocloud.net/tools/ani [27]). The resistance gene identifier (RGI) module of the comprehensive antibiotic resistance database was used to find and annotate antimicrobial resistance genes [28]. The functional pathways were inferred using GhostKOALA-KEGG Mapper [29,30]. The dbCAN3 was used to annotate carbohydrate-active enzymes (CAZymes) [31].

## Results and discussion

**Fig. 1.**
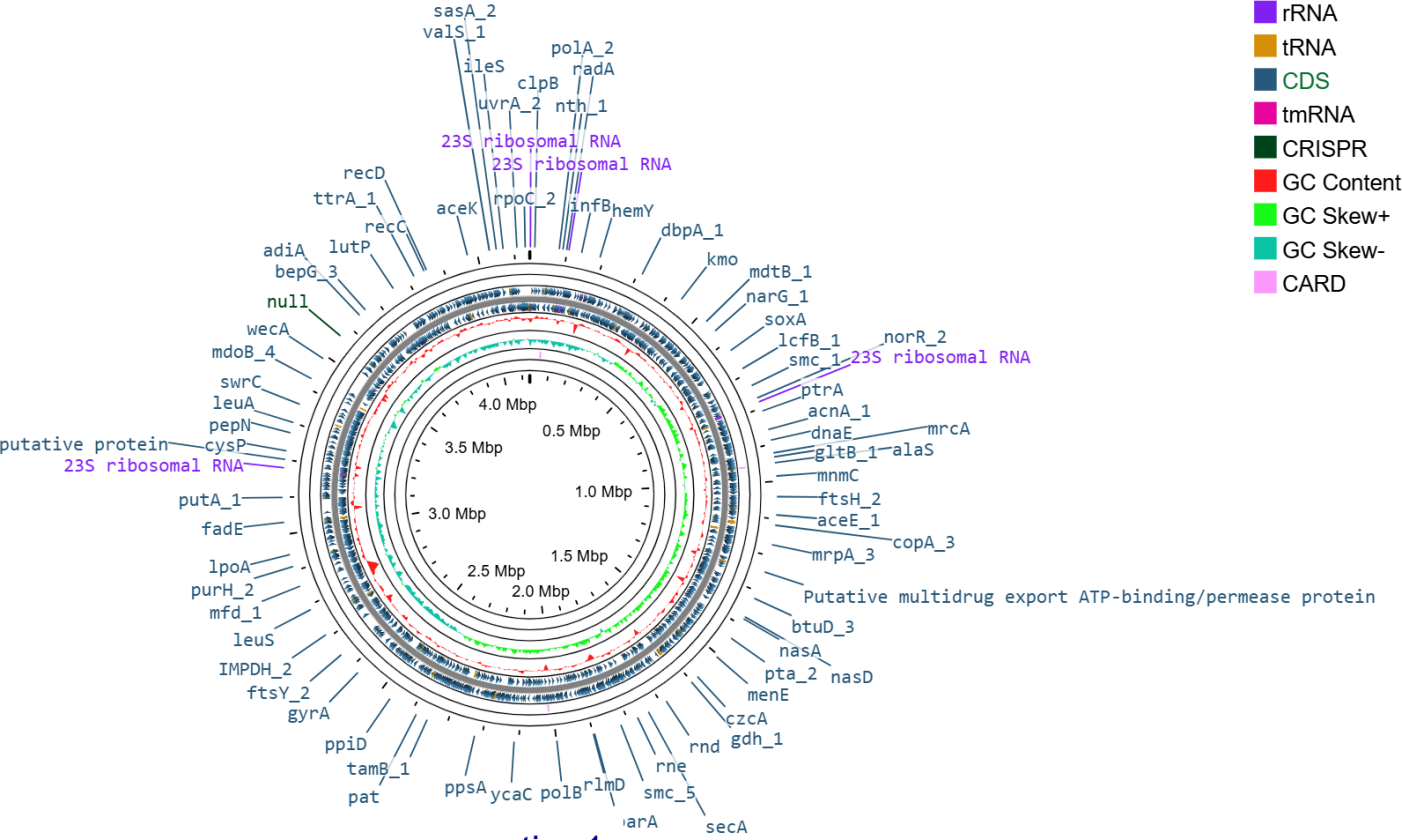
A circular genome map of *Billgrantia* sp. strain SL18_1 (representation only) along with the identified genes, G+C content and GC skew (+/).

**Fig. 2.**
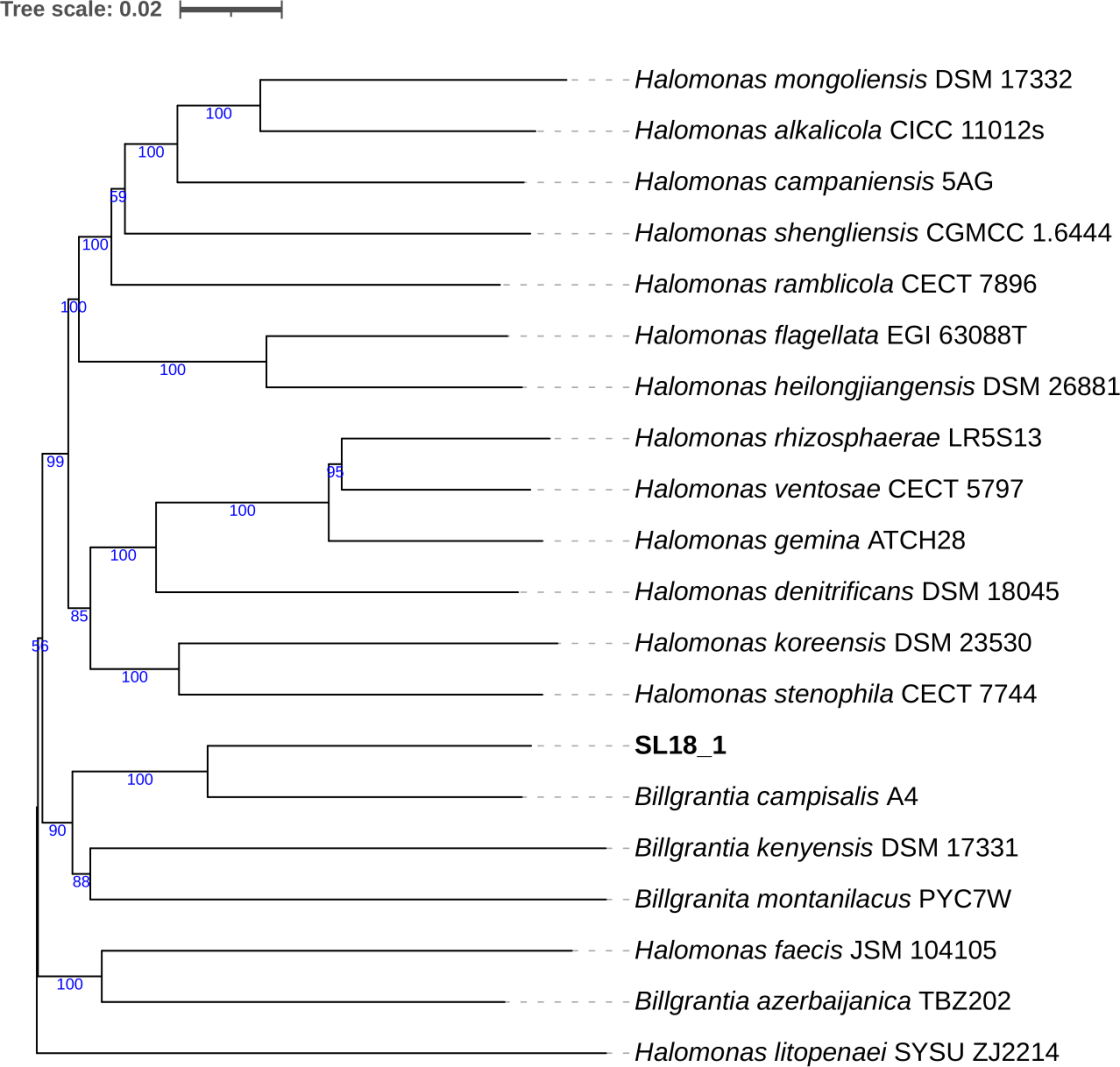
Phylogram showing the phylogenetic relation of *Billgrantia* sp. strain SL18_1 with other known species of *Halomonas*. The numbers in blue represent the bootstrap values for each clade. All information has been obtained from the TYGS server.

The isolated bacterium with strain number SL18_1 is Gram-negative and rod-shaped. Based on blastn, the 16S rRNA sequence of this isolate show 99.3% identity and 100% query coverage with *Billgrantia chromatireducens* AGD 8-3^T^. The draft genome size is 4.1 Mbp and the assembly consist of one contig (Fig. 1). The G+C percentage is 66.27% with the presence of 4,482 CDS (Table 1). The genome is 93.65% complete with only 1.9% contamination. Based on genome-based distance phylogeny, the closest relative of strain SL18_1 is *Billgrantia campisalis* A4^T^, placed into a separate clade with strong bootstrap support of 100 (Fig. 2). The Digital DNA-DNA Hybridization (dDDH) values for SL18_1 with *B. campisalis* A4^T^ are 34.5%, the OrthoANI value is87.69 and the AAI percentage (AAI%) is 84.7%. The difference in G+C content is only 0.02 mol% (Table 2). All these values are below the suggested cutoff for species delineation, suggesting the strain is a potential novel species within the genus *Billgrantia*. The GGDC between *B. campisalis* A4^T^ and strain SL18_1 has been calculated to be 0.12. The genome is found to code for 12 rRNA, 67 tRNA and 1 tmRNA. The *in silico* phenotyping indicated the strain SL18_1 to be aerobic, motile and Gram-negative (Fig. S1 available in the online Supplementary Material). The isolate is predicted to hydrolyse urea, can convert nitrate to nitrite and can grow on MacConkey as well as ordinary blood agar. Based on phenotyping (*in silico*), the isolate is predicted to exhibit catalase and oxidase activity while being negative for methyl red (MR) test, Voges–Proskauer (VP) test, citrate utilization, hydrogen sulphide (H₂S) and indole production. The biochemical tests for some of these properties such as indole, VP, MR, catalase and oxidase were undertaken using the isolate and have been found to be negative. The phenotyping analysis indicates that the isolate may not be able to utilize most of the complex carbohydrates and other substrates, including gelatin, starch, lactose, casein, xylose, salicin, raffinose, d-rhamnose and d-mannitol. This is also supported by the dbCAN results, where only one CAZyme, maltodextrin glucosidase, has been identified within the GH13 glycoside hydrolase family, and one hit correspond to GH3 family enzyme beta-*N*-acetylglucosaminidase, a single hit for GH73 peptidoglycan hydrolase and two hits with membrane-bound lytic murein transglycosylase B. No polysaccharide lyases and cellulosome hits have been identified from the draft genome data.

**Table 1.** Statistics of the draft genome representing *Billgrantia* sp. strain SL18_1

**Feature**	**Value**
Genome size (bp)	4,141,733
Number of contigs	1
Largest contig (bp)	4,141,733
Total length (bp)	4,141,733
N50	4,141,733
L50	1
G+C (mol%)	66.27
CDS	4,482
rRNA	12
tRNA	67
tmRNA	1

**Table 2.** Comparison of strain SL18_1 with all reported genomes belonging to the genus *Billgrantia*

Closest relative	dDDH (%)	AAI (%)	G+C (mol%)	OrthoANI (%)	Difference G+C (%)	GGDC
*B. campisalis* A4^T^	34.5	84.7	66.28	87.69	0.02	0.1205
*Halomonas alkalicola* CICC 11012s^T^	26	–	68.21	82.35	1.29	0.1688
*Halomonas campaniensis* 5AG^T^	25.5	84.1	68.71	82.56	2.45	0.1703
*Halomonas mongoliensis* DSM 17332^T^	25.3	–	67.71	81.83	1.46	0.1720
*Halomonas heilongjiangensis* DSM 26881^T^	25.2	84.7	66.08	81.87	0.41	0.1735
*Halomonas ramblicola* CECT 7896^T^	25.2	–	67.87	81.71	1.62	0.1729

The genes namely, *ureA*, *ureB* and *ureC* coding for the gamma, beta and alpha subunits of the urease enzyme as part of the urea cycle have been identified within the genome. Additionally, *ureF*, *ureG* and *ureD* coding for the urease accessory protein are also present within the genome. Based on *in silico* analysis, the isolate is predicted to convert nitrate to nitrite. Furthermore, the presence of genes involved in nitrogen metabolism indicates potential ability of this isolate to reduce nitrate indicative of functional linkages to denitrification process. The gene *napA*, which codes for periplasmic nitrate reductase, and *narG* and *narY*, encoding nitrate reductase, are also present within the genome. For the nitrate reduction pathway, *nirB*, *nirD* and *nasD*, coding for nitrite reductase, have been identified. Genes involved in the denitrification process included *nirS*, encoding for nitrite reductase, which converts nitrite to nitric oxide. Besides, *norB* and *norC*, coding for nitric-oxide reductase, which is involved in the conversion of nitric oxide to nitrous oxide, have been also identified. Within the genome, *nosZ*, coding for nitrous-oxide reductase, which converts nitrous oxide to nitrogen, has been also identified.

The motility of strain SL18_1 is supported by the presence of a fairly complete set of genes responsible for flagellar assembly and function within the genome. The genes such as *fliP*, *fliQ*, *fliR*, *flhA* and *flhB*, coding for flagellar biosynthesis proteins, are also present. Other genes such as *flgA*, *flgB*, *flgC*, *flgF* and *flgG* encoding for flagellar basal body rod proteins have been identified. Besides, *flgH* and *flgI* responsible for forming the l- and P-rings, respectively while motor switch proteins, such as *fliG* and *fliM*, coding for flagellar motor switch proteins, have been also identified within the genome. Additionally, *fliF*, which encodes the M-ring protein, has been identified along with *fliE* and *fliD*, suggesting the hook-basal body complex is intact. The presence of *flaA*, likely encoding flagellin, supports the formation of the filament itself. On the regulatory side, *flhC* and *flhD* (flagellar transcriptional regulators) have been identified, pointing towards a well-regulated flagellar system. While experimental validation is still needed, these findings indicate that the isolate likely exhibits flagellar-based motility.

In the genome of this isolate, KEGG pathway module showed the presence of complete pathway linked to degradation of benzoate compounds. The genes involved in osmoregulation and acid stress tolerance have been identified within the genome. This strain possesses specific osmoregulatory genes, possibly allowing the bacterium to grow under high osmotic stress. These genes include *osmW*, *osmY* (encoding osmoprotectant import permease protein); *osmX*, *osmV* (encoding osmoprotectant import ATP-binding protein); and *opuE* (encoding osmoregulated proline transporter). The presence of *ibaG* gene indicates the ability of this isolate to cope with acidic stress. Based on antiSMASH results, ectoine biosynthesis gene cluster was identified. In addition, AntiSMASH analysis resulted in the identification of a putative siderophore BGC within the genome. The cluster showed homology to the siderophore biosynthetic protein *IucA/IucC*. Ectoine acts as a protector against osmotic stress and enables bacterial cell to survive within high salt environment. The genome of strain SL18_1 also possesses genes for multidrug resistance proteins, including *mdtA*, *mdtB*, *mdtK* and *norM*. Based on RGI results, the strain can exhibit resistance against fluoroquinolone, diaminopyrimidine, phenicol and phosphonic acid antibiotics. Overall, *Billgrantia* sp. strain SL18_1 could have potential application in tackling excess nitrogen load across ecosystems but will require robust validation to assess long-term efficacy based on bioremediation.

## Supplementary material

10.1099/acmi.0.001093.v3 Supplementary Material 1.

## Abbreviations

AAI: average amino acid identity
ANI: average nucleotide identity
BGCs: biosynthetic gene clusters
CAZyme: carbohydrate-active enzyme
dDDH: Digital DNA-DNA Hybridization
gDNA: genomic DNA
MR: methyl red
RGI: resistance gene identifier
TYGS: type (strain) genome server
VP: Voges–Proskauer
