# Supplementary material for: Genome description of a potentially novel species of Billgrantia sp. strain SL18_1 isolated from Sambhar Lake, India
Source: Access Microbiol. 2026 Jun 25;8(6):001093.v3. doi: 10.1099/acmi.0.001093.v3 (PMC13297067; doi:10.1099/acmi.0.001093.v3)
Supplement: Supplementary Material 1. [file acmi-8-01093-s001.pdf]

Supplementary Figure and Table

Fig.S1: *In silico* phenotyping characters of *Billgrantia* sp. strain SL18_1

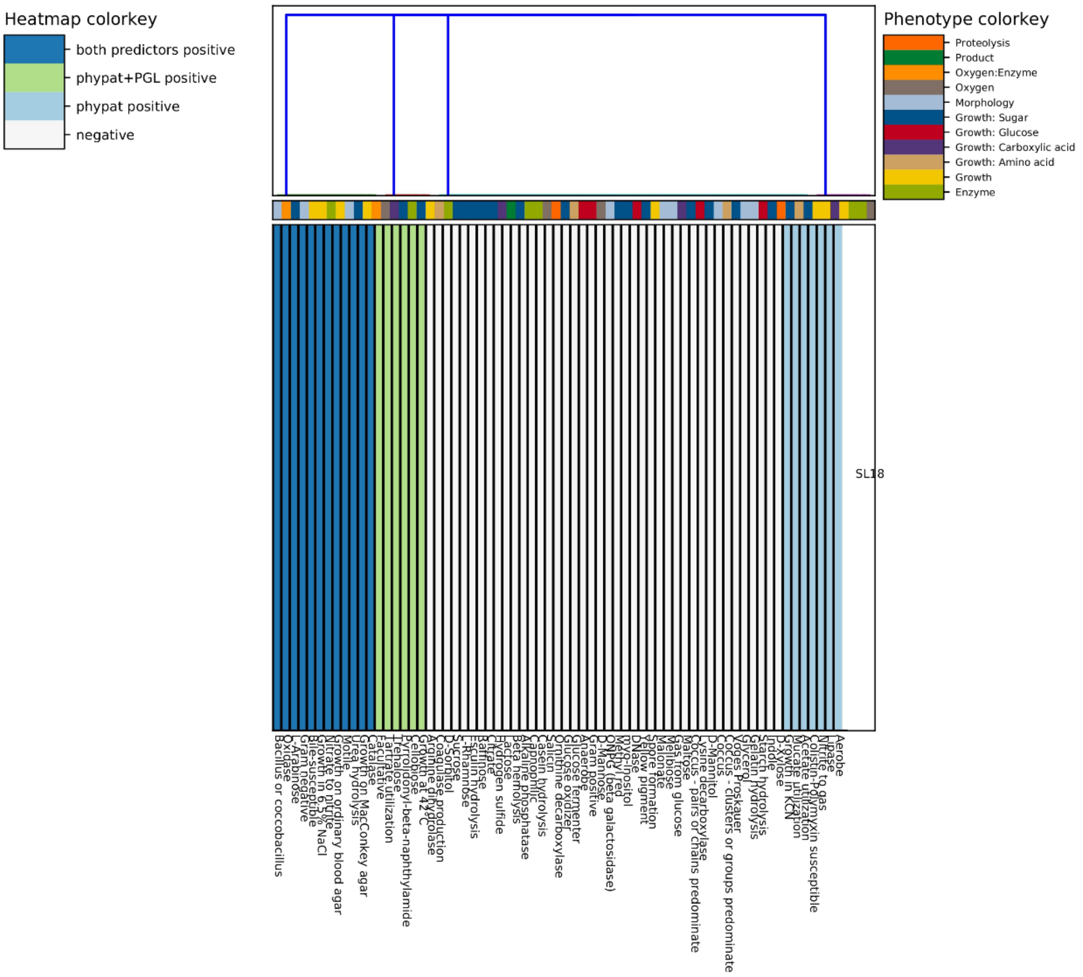

Table S1: Nucleotide sequences of the primers used for 16S rRNA amplification of *Billgrantia* sp. strain SL18_1

**Primer**	**Sequence**
FC27	5′-AGAGTTTGATCCTGGCTCAG-3′
RC1492	5′-TACGGCTACCTTGTTACGACTT-3′
